# Extra-Adrenal Retroperitoneal Myelolipoma Resected by Laparoscopy in an Asymptomatic Patient

**DOI:** 10.1155/2021/8849194

**Published:** 2021-03-13

**Authors:** Gabriel Ambrogi, Maria Madureira Murta, André Silva Guimarães Moeda, Ramon Brandão Barbosa, Israel Rodrigues da Silva, Ana Beatriz Tabosa Negrão Xavier, Matheus Vieira dos Santos, Lucas Martinucci de Oliveira, Bernardo Luiz Campanário Precht, Renato Panhoca, Luis Augusto Seabra Rios

**Affiliations:** ^1^General Surgery Resident of the General Surgery and Oncology Department of the Hospital do Servidor Público Estadual (HSPE), The Medical Sciences Course, Faculty of Health Sciences, Metropolitan University of Santos, Santos, SP, Brazil; ^2^General Surgery Resident of the General Surgery and Oncology Department of the Hospital do Servidor Público Estadual (HSPE), The Medical Sciences Course, Faculty of Health Sciences, Fluminense Federal University, Rio de Janeiro, RJ, São Paulo, Brazil; ^3^General Surgery Resident of the General Surgery and Oncology Department of the Hospital do Servidor Público Estadual (HSPE), The Medical Sciences Course, Faculty of Health Sciences, University of Volta Redonda, Rio de Janeiro, RJ, São Paulo, Brazil; ^4^General Surgery Resident of the General Surgery and Oncology Department of the Hospital do Servidor Público Estadual (HSPE), The Medical Sciences Course, Faculty of Health Sciences, State University of Maringá, Paraná, PR, São Paulo, Brazil; ^5^General Surgery Resident of the General Surgery and Oncology Department of the Hospital do Servidor Público Estadual (HSPE), The Medical Sciences Course, Faculty of Health Sciences, University of the City of São Paulo, Santos, SP, Brazil; ^6^Urology Resident of the Hospital do Servidor Público Estadual (HSPE), The Medical Sciences Course, Faculty of Health Sciences, Evangelical Faculty of Paraná, Paraná, PR, São Paulo, Brazil; ^7^Urology Resident of the Hospital do Servidor Público Estadual (HSPE), The Medical Sciences Course, Faculty of Health Sciences, University of Ribeirão Preto, São Paulo, SP, Brazil; ^8^Urology Resident of the Hospital do Servidor Público Estadual (HSPE), The Medical Sciences Course, Faculty of Health Sciences, Fluminense Federal University, Rio de Janeiro, RJ, São Paulo, Brazil; ^9^Department of Urology of the Hospital do Servidor Público Estadual (HSPE), The Medical Sciences Course, Faculty of Health Sciences, Federal University of Uberlandia, Minas Gerais, MG, São Paulo, Brazil; ^10^Department of Urology at Hospital do Servidor Público Estadual (HSPE), The Medical Sciences Course, Faculty of Health Sciences, Pontifical Catholic University of São Paulo, Sorocaba, SP, Brazil

## Abstract

Myelolipomas are rare benign neoplasms that commonly develop in the adrenal glands. Less frequently, they can affect other organs such as the liver, stomach, liver, lung, and retroperitoneum. It affects more women, with an average age of around 61 years. Histologically, they are composed of mature adipose tissue and hematopoietic cells. With the evolution of immunohistochemistry, there are characteristics that can differentiate from malignant tumors such as liposarcomas. Its treatment remains based on surgical resection and long-term outpatient follow-up.

## 1. Introduction

Myelolipomas are uncommon, benign tumors. These are generally solitary and nonfunctioning neoplasms, consisting of mature fat tissue associated with hematopoietic cells that are similar to those found in the bone marrow [1].

In the majority of cases, myelolipomas are located in the adrenal glands. They are rarely found, among other sites, in the retroperitoneum, mediastinum, lung, liver, and stomach [2].

The disease frequently affects the fifth and sixth decade of life [3]. Our case report describes a patient developing a progressive, asymptomatic left perirenal mass during follow-up of urology treatment, which was laparoscopically resected.

## 2. Case Report

**Figure 1:**
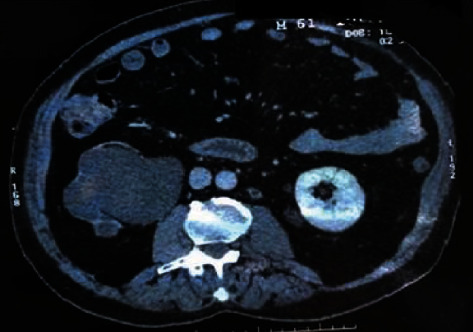
CT of June 2, 2016 showing posteromedial oval image to the left kidney of 2,4 cm.

FO, a 64 years old, Caucasian male, follow-up of lower urinary tract symptoms (LUTS) and nonfunctioning kidney due to a chronic ureteropelvic junction obstruction confirmed by DMSA scintigraphy in our service, underwent an abdominal computed tomography (CT) scan in July 2016. In addition to the common abdominal imaging findings, the abdominal CT scan showed an oval image of densified posteromedial fat attenuation in the left kidney (Figure 1).

**Figure 2:**
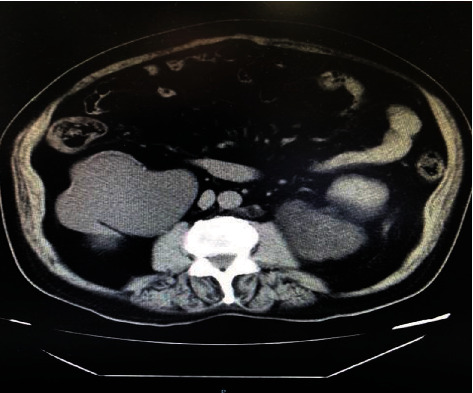
CT of November 2019 showing the important growth of the mass located between the left kidney and the psoas muscle.

In 2017, the patient underwent prostate transurethral resection for benign prostatic hyperplasia (BPH). He has remained asymptomatic since that time. During an outpatient follow-up in 2019, a new abdominal CT detected a significant increase in mass size, measuring 7.1 x 5.0 x 7.0 cm, without evidence of suspicious lymphadenopathy (Figure 2).

The emergence of this abnormality led to retroperitoneal mass resection by laparoscopy transperitoneal approach. The surgical specimen was submitted to histopathology for examination (HP).

In the immediate postoperative period, the patient was admitted to the intensive care unit (ICU) for 24 hours in a hemodynamically stable condition without complications. He was then sent to the ward for two more days, where he remained in a satisfactory condition until hospital discharge. The patient currently does not show any signs of tumor recurrence.

**Figure 3:**
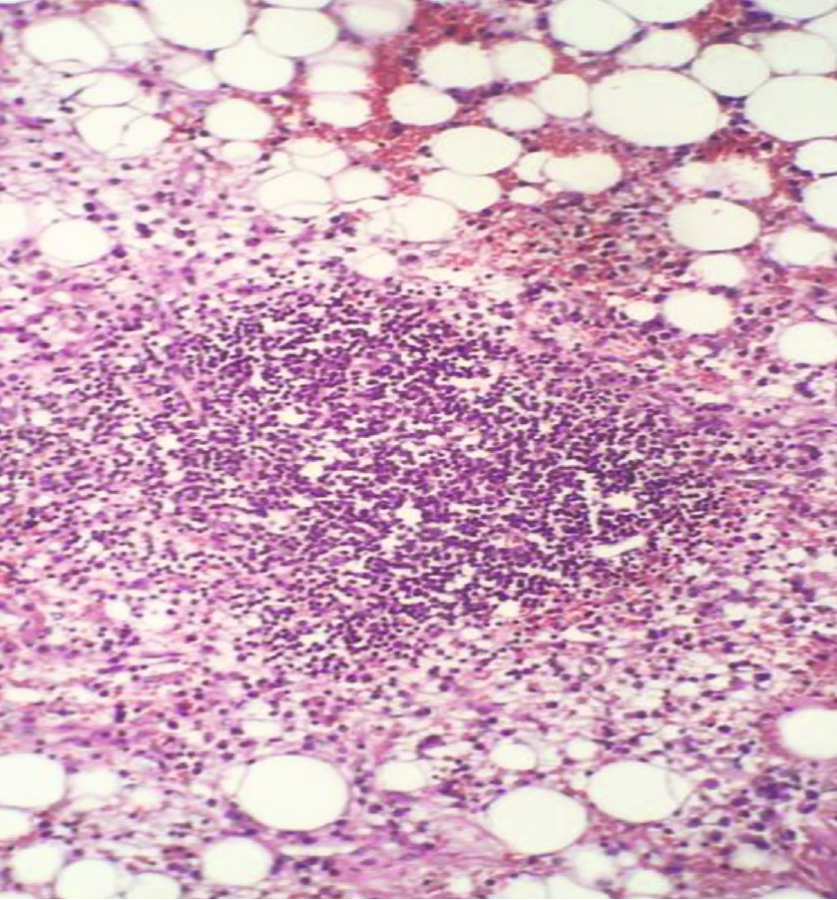
Mature adipose tissue associated with a mixed inflammatory infiltrate with sparse hematopoietic precursors, lymphocytes, and plasmacytes. Hematoxylin Eosin (HE) 100 X.

**Figure 4:**
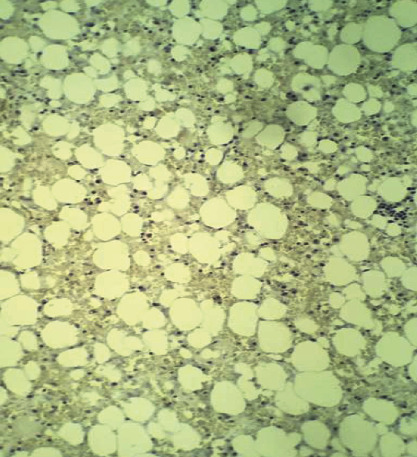
Immunohistochemistry showing the absence of expression of the MDM2 protein, CDK4.

HP and immunohistochemistry results showed the presence of mature adipose tissue, associated with mixed inflammatory infiltrates and hematopoietic precursors, in addition to amplification of MDM2 and CDK4 gene products, suggestive of myelolipoma (Figures 3 and 4).

## 3. Discussion

Myelolipomas are neoplasms of unknown origin, varying in size from 2 to 26 cm. In most cases, myelolipomas are asymptomatic, although larger tumors can cause a mass effect or hemorrhage [4]. Extra-adrenal myelolipoma predominates in females (2 : 1) and are found in middle-aged individuals and in the elderly (mean age, 61 years) [5]. The reported incidence of the tumor ranges from 0.08% to 0.4% [6] at autopsy.

Diagnosis of these tumors prior to resection, surgery, and/or biopsy is difficult and requires histopathological analysis. In tomographic and/or magnetic resonance imaging, differentiation between myelolipomas and well-differentiated liposarcomas in the retroperitoneum is often not possible [4]. In addition to liposarcomas, differential diagnoses of this rare disease include hematopoietic tumors and/or extramedullary hematopoiesis, due to the presence of hematopoietic precursor cells [7–8]. In the retroperitoneum, more than 50% of myelolipomas are located in the presacral region, different from our case which originated in the perirenal region [9].

Histologically, myelolipomas can be differentiated from liposarcomas and focal mass-forming extramedullary hematopoiesis. Foci of extramedullary hematopoiesis, e.g., myeloproliferative diseases, hemolytic anemia, and severe skeletal disease exhibit a smaller amount of fat tissue and poorly defined lesions, while myelolipomas are well-defined lesions, composed of varying amounts of mature adipose tissue with cells arising from the bone marrow. Liposarcomas present lipoblasts, zones of cellular atypia, and are less frequently marginalized [11]. Immunohistochemistry currently points to the diagnosis of liposarcoma by detection of MDM2 and CDK4 protein expression. In contrast, myelolipomas do not express these proteins [12–13].

Treatment still relies on surgical resection. Tumor recurrence is infrequent. With the wider use of immunohistochemistry, these lesions can be more clearly differentiated. However, in settings where access to this resource is unavailable, misdiagnoses may occur. Retroperitoneal malignancies such as liposarcomas can remain undiagnosed, drastically worsening patient prognosis.
